# Vaccine Knowledge, Attitude, and Hesitancy Among Healthcare Professionals in the Kingdom of Bahrain: A Cross-Sectional Survey

**DOI:** 10.7759/cureus.82519

**Published:** 2025-04-18

**Authors:** Safiya AlMusawi, Marya Radhi, Thrshith Mani Prabu Kumar, Eman AlSalman, Mohammed Khaleel, Khalil Altaitoon, Fatema Majeed, Zakeya AlMusawi

**Affiliations:** 1 Medical Microbiology, Salmaniya Medical Complex, Manama, BHR; 2 General Medicine, Royal College of Surgeons in Ireland, Isa Town, BHR; 3 School of Medicine, Royal College of Surgeons in Ireland, Manama, BHR; 4 Family Medicine, Primary Healthcare Centers, Manama, BHR; 5 Nursing, Primary Healthcare Centers, Manama, BHR; 6 Nursing, Salmaniya Medical Complex, Manama, BHR

**Keywords:** attitude, healthcare professionals, hesitancy, knowledge, vaccination

## Abstract

Background

Healthcare professionals (HCPs) are the cornerstone of any successful vaccination program. Their knowledge, attitude, and hesitancy are major factors in vaccination uptake.

Aim

We sought to study the knowledge, attitude, and hesitancy towards the recommended vaccination among healthcare professionals in the Kingdom of Bahrain.

Methods

A cross-sectional survey was conducted among HCPs at governmental hospitals and primary healthcare centers in the Kingdom of Bahrain. HCPs completed a standardized questionnaire online or in person related to their vaccine knowledge and attitude. Data were weighted for different demographic characteristics. To assess vaccine hesitancy, 15 questions related to vaccine-related attitudes and self-vaccination attitudes were used. Answers to the questions were scored, and the scoring was categorized as low hesitancy, moderate hesitancy, and high hesitancy.

Results

The study included 552 HCPs. The majority of the participants (75%) believed that vaccines are safe, and 85.5% believed that vaccines protect against infectious diseases. Hepatitis B vaccine and varicella vaccine uptake were less compared to other types of vaccine. None or low vaccine hesitancy was found in 75.2% of the HCPs. Long-term side effects were found to be the highest concern about vaccination in our participants, with 48.4%. 72.5% of participants had not received any training related to vaccination, and 44.1% of those who did had their last training more than 3 years ago.

Conclusions

Interventions are required to help hesitant HCPs adopt more proactive vaccination practices, and the implementation of policies to check the vaccination status and training is needed urgently.

## Introduction

Vaccination is proven to be the most cost-effective public health measure that plays a major role in reducing morbidity and mortality associated with various infectious diseases [1-2]. Despite the continuous regulatory monitoring of vaccine safety by the World Health Organization (WHO) and other organizations, critics and hesitancy about vaccines exist in communities globally [3].

In 2019, the WHO listed vaccine hesitancy as one of the top ten threats to global health [4]. The definition of vaccine hesitancy is complex and varies in the literature. A group of experts defines vaccine hesitancy as the behaviors, beliefs about vaccine safety and efficacy, attitudes about mandates, and trust towards vaccines [5]. According to WHO's vaccine advisory group, the factors that lead to vaccine hesitancy include complacency and inconvenience in accessing vaccines and lack of confidence [4]. A major factor affecting vaccine hesitancy is a lack of confidence, which is a major cause of low vaccination rates in the community [3].

Vaccine hesitancy is found to have increased to an alarming level in the last few years at the public level as well as among healthcare professionals (HCPs), especially after the introduction of the severe acute respiratory syndrome coronavirus 2 (SARS-CoV-2) vaccine [6-7]. Like the general public, healthcare professionals' behaviors toward vaccination may be influenced by their knowledge, emotions, beliefs, and attitudes​​​​​​ [6]*. *HCPs are the cornerstone of vaccination [8-9]. They motivate their patients to be vaccinated, answer their questions and concerns about vaccines, and because their patients trust them strongly, more than any other source of information, they are the most trusted advisors and influencers of vaccine decisions [6, 8, 9]. Same as in the community, evidence has been found that low confidence in vaccines among HCPs has been associated with low uptake rates of vaccines [10-11]. Also, many studies found that a strong recommendation from HCPs to patients about vaccination is likely to increase uptake of vaccination [12-15]. Moreover, HCPs who have themselves been vaccinated are found to be more likely to be knowledgeable about vaccination, more effective in communicating and improving public confidence, and demand for vaccination [16]. Their attitudes and knowledge towards vaccinations and preparedness with adequate training were found to be important factors in handling difficult conversations, especially with those who demonstrate reluctance or hesitancy towards vaccination [17]. Although they can move undecided populations not only towards vaccination but also towards non-vaccination [6]. In an era of rising vaccine hesitancy, understanding HCPs' vaccine confidence is essential.

The aim of this study is to explore the knowledge, attitude, and hesitancy towards recommended vaccination among healthcare professionals in the Kingdom of Bahrain.

## Materials and methods

We conducted an anonymous online survey and a cross-sectional in-person questionnaire for healthcare professionals at government hospitals and primary healthcare centers in the Kingdom of Bahrain. The survey was started in July 2024 and ended in September 2024. The online survey was distributed via email and WhatsApp. Participants were required to provide a response to every item in the survey, and confidentiality was assured and stated clearly at the beginning of the questionnaire. Doctors, nurses, and laboratory workers were the only healthcare professionals included in this survey. We excluded other healthcare workers, other institutes, incomplete or partial responses, and duplicates from the analysis.

Survey questions were developed according to the recommended vaccination for healthcare workers in the Kingdom of Bahrain. A pilot test survey was sent to other experts in the field to assess the questions prior to sending the survey to participants. Questions were divided into three sections: background information, knowledge of vaccination, and attitude. The questionnaire asked about participants’ characteristics (sex, age, nationality, educational level, occupation, duration of working in their profession, and name of health facility), the knowledge about the vaccines that the healthcare workers should receive, and the doses of vaccinations they received against Hepatitis B, seasonal influenza, measles-mumps-rubella (MMR), tetanus-diphtheria (Td), varicella, meningococcal conjugate, and severe acute respiratory syndrome coronavirus 2 (SARS-CoV-2) vaccines. It also asked if their knowledge about vaccination is insufficient or outdated, and if they have received training or participated in courses on vaccination related to healthcare workers. The attitude section included questions related to their belief about whether vaccines are safe, if they are worried about the side effects, their greatest concern about vaccines, if they are following their vaccinations in a regular manner, the last time they visited the clinic to follow up their vaccination, if they support mandatory occupational vaccination, if they encourage their patients to receive vaccines, and why they think that the vaccination is important.

Statistical analysis

Data was analyzed by using the Statistical Package for the Social Sciences (SPSS) version 26.0 (IBM Corp., Armonk, USA). Frequencies and percentages were calculated for the qualitative variables such as gender, age groups, nationality, occupation, duration of work, and institute. Percentages were also calculated for knowledge and attitude questions. For vaccine hesitancy, 15 questions related to vaccine-related attitudes and self-vaccination attitudes were used to assess hesitancy. Each correct answer was scored as one (1), while incorrect or "Not Sure" responses were scored as zero (0), resulting in a total score range of 0 to 15. The scoring was categorized as follows: 0 to 4 indicated almost none to low hesitancy, 5 to 8 indicated moderate hesitancy, and scores above 8 indicated high hesitancy. Due to only 13 cases falling into the high hesitancy category, they were merged with the moderate hesitancy group. All the demographic, knowledge, and attitude-related questions were compared between the two groups (almost none tolow hesitancy vs. moderate to high hesitancy) by using the Chi-square test. Logistic regression was used to calculate relative risk for predictors like nationality, institute, knowledge, and perception about vaccines. P-value ≤ 0.05 was considered significant.

## Results

A total of 485 HCPs participated in the online survey, and 124 participated in the in-person questionnaire. Only 552 HCPs were included in the analysis. The majority of HCPs were female (447, 81%), while 105 (19%) were male. The largest age group was 31-40 years, 273 (49.5%), followed by 20-30 years, 174 (31.5%). A total of 516 (93.5%) of the participants were Bahraini, while 6.5% were non-Bahraini. Most participants held a bachelor's degree (248, 44.9%), followed by a medical degree (136, 24.6%). Nurses made up the largest group, 252 (45.7%), followed by doctors, 200 (36.2%), and laboratory workers, 100 (18.1%). Two hundred and ninety-nine (54.2%) had worked for less than 10 years, while 253 (45.8%) had worked for more than 10 years. Three hundred (54.3%) worked in primary care, and 252 (45.7%) worked in governmental hospitals. The majority worked in the nursing department (252, 45.7%), followed by family medicine (79, 14.3%) and pathology departments (63, 11.4%) (Table 1).

**Table 1:** Characteristics of the participating healthcare professionals (n = 552)

		Frequency	Percentage
Sex	Male	105	19
	Female	447	81
Age	20 – 30 years	174	31.5
	31 - 40 years	273	49.5
	41 - 50 years	82	14.9
	51 - 60 years	20	3.6
	61 - 70 years	3	0.5
Nationality	Bahraini	516	93.5
	Non-Bahraini	36	6.5
Education	Diploma degree	92	16.7
	Bachelor degree	248	44.9
	Master degree	61	11.1
	Medical degree	136	24.6
	Others	15	2.7
Occupation	Doctor	200	36.2
	Nurse	252	45.7
	Laboratory worker	100	18.1
Duration of Work	< 10 years	299	54.2
	> 10 years	253	45.8
Institute	Primary Care	300	54.3
	Governmental hospitals	252	45.7
Department	Nursing	252	45.7
	Family physician	79	14.31
	Pathology	63	11.4
	Laboratory worker (Primary care)	48	8.70
	Internal Medicine	23	4.17
	General practice	13	2.36
	Gynecology-obstetrics	8	1.45
	Anesthesia	7	1.27
	Cardiology	5	0.91
	Ear-Nose-Throat	5	0.91
	Pediatrics	5	0.91
	Psychiatry	5	0.91
	Nephrology	4	0.72
	Ophthalmology	4	0.72
	Radiology	4	0.72
	Oncology	3	0.54
	Surgery	3	0.54
	Dermatology	2	0.36
	Intensive care unit	2	0.36
	Emergency	1	0.18
	Genetics	1	0.18
	Hematology	1	0.18
	Neurology	1	0.18
	Orthopedics	1	0.18
	Plastic surgery	1	0.18
	Urology	1	0.18
	Not mentioned clearly	10	1.81

The majority of the HCPs believed that they should receive vaccines such as influenza (82.9%), MMR (80.8%), Td (80.43%), meningococcal conjugate (76.63%), and SARS-CoV-2 (71.38%), with Hepatitis B having the highest support (97.83%). The majority of the HCPs, 283 (51.3%), received three doses of the hepatitis B vaccine, followed by two doses (149, 27%), while 4% never received the vaccine. A total of 321 (58.2%) received two doses of the MMR vaccine, and 5.3% never received the vaccine. The majority of 356 (64.5%) HCPs received a single dose of influenza vaccine annually, while 16.8% never received the vaccine. The tetanus-diphtheria vaccine was taken by 365 (66.1%) HCPs every 10 years, and 6% never received the vaccine. The majority, 308 (55.8%), never received the varicella vaccine, while 21.2% received one dose and only 1.3% received three doses. A total of 333 (60.3%) participants received one dose and booster every 5 years of meningococcal conjugate vaccine, and 19.2% never received the vaccine. While the majority (467, 84.6%) of participants received 1-3 doses of the SARS-CoV-2 vaccine, 2.9% never received the vaccine.

Regarding knowledge of vaccines and vaccinations, 219 (39.7%) were unsure if their knowledge was insufficient or outdated, and only 183 (33.2%) participants reported that they have good knowledge of vaccines. The majority of participants had not received any training related to vaccination (400, 72.5%), and 67 (44.1%) of those who did had their last training more than 3 years ago. While 396 (71.7%) were willing to participate in informative sessions about vaccination, 12.1% were not (Table 2).

**Table 2:** Experience, knowledge, and opinions of healthcare professionals related to vaccines and vaccination (n = 552)

		Frequency	Percent
Received hepatitis B vaccine, dose(s)	One dose	44	8
	Two doses	149	27
	Three doses	283	51.3
	Four doses	54	9.8
	Never received	22	4
Received measles-mumps-rubella (MMR) vaccine, dose(s)	One dose	118	21.4
	Two doses	321	58.2
	Three doses	57	10.3
	Four doses	27	4.9
	Never received	29	5.3
Received influenza vaccine, dose(s)	Single dose annually	356	64.5
	Single dose every 5 years	76	13.8
	Single dose every 10 years	27	4.9
	Never received	93	16.8
Received tetanus-diphtheria (Td) vaccine every 10 years	Yes	365	66.1
	No	154	27.9
	Never received	33	6
Received varicella vaccine, dose(s)	One dose	117	21.2
	Two doses	115	20.8
	Three doses	7	1.3
	Four doses	5	0.9
	Never received	308	55.8
Received meningococcal conjugate vaccine, dose(s)	One dose and booster every 5 years	333	60.3
	Two doses and booster every 5 years	88	15.9
	Three doses and booster every 5 years	18	3.3
	Four doses and booster every 5 years	7	1.3
	Never received	106	19.2
Received SARS-CoV-2 vaccine, dose(s)	1 - 3	467	84.6
	> 3	69	12.5
	Never received	16	2.9
Knowledge of vaccines and vaccination is insufficient or outdated	Yes	150	27.2
	No	183	33.2
	Not Sure	219	39.7
Participation in training or courses on vaccination related to healthcare workers	Yes	152	27.5
	No	400	72.5
Duration of last training on vaccination related to healthcare worker	Less than 1 year ago	53	34.9
	1-3 years ago	32	21.1
	More than 3 years ago	67	44.1
Willing to participate in an informative session about vaccines	Yes	396	71.7
	No	67	12.1
	Not Sure	89	16.1
Reason for not willing to participate in an informative session about vaccines	Don’t have time	33	49.3
	Never heard of such sessions	7	10.4
	Not interested	24	35.8
	Don’t trust vaccines	3	4.5

Attitude toward the vaccine is presented in Table 3. Three-fourths of the participants believed vaccines are safe (414, 75%), while 116 (21%) were unsure. A total of 195 (35.3%) were worried about short-term side effects of the vaccine, while 312 (56.5%) were worried about long-term side effects. Long-term side effects (267, 48.4%) and vaccine safety (227, 41.1%) were the top concerns about the vaccine. 371 (67.2%) participants followed their vaccinations regularly, 439 (79.5%) participants supported mandatory occupational vaccination, and 324 (58.7%) HCPs always encouraged patients to receive the vaccine. While 472 (85.5%) believed vaccines protect against diseases, and 413 (74.8%) believed that vaccines prevent epidemics, only 15 (2.7%) participants believed that vaccines are not important.

**Table 3:** Attitude of healthcare professionals towards vaccines and vaccination (n = 552)

		Frequency	Percent
Believe that the vaccine is safe	Yes	414	75
	No	22	4
	Not Sure	116	21
Worry about short-term side effects	Yes	195	35.3
	No	314	56.9
	Not Sure	43	7.8
Worry about long-term side effects	Yes	312	56.5
	No	180	32.6
	Not Sure	60	10.9
Greatest concern about vaccine (select all that apply)	Short-term side effects	91	16.5
	Long-term side effects	267	48.4
	Vaccine efficacy	134	24.3
	Vaccine safety	227	41.1
	Don't have any concern	134	24.3
Regular follow-up of the vaccinations	Yes	371	67.2
	No	181	32.8
Last time they visited the clinic to follow up on vaccination	Less than 1 year ago	285	51.6
	1-3 years ago	153	27.7
	More than 3 years ago	86	15.6
	Never	28	5.1
Support mandatory occupational vaccination	Yes	439	79.5
	No	113	20.5
Encourage their patients to receive vaccines	Always	324	58.7
	Sometimes	166	30.1
	Never	19	3.4
	No direct involvement with patients	43	7.8
Opinion about the importance of vaccines	Protect us from diseases	472	85.5
	Protect vulnerable individuals	365	66.1
	Prevent epidemics and pandemics from occurring	413	74.8
	Promote public health	405	73.4
	Prevent long-term complications of diseases	339	61.4
	Don't think they are important	15	2.7

**Figure 1:**
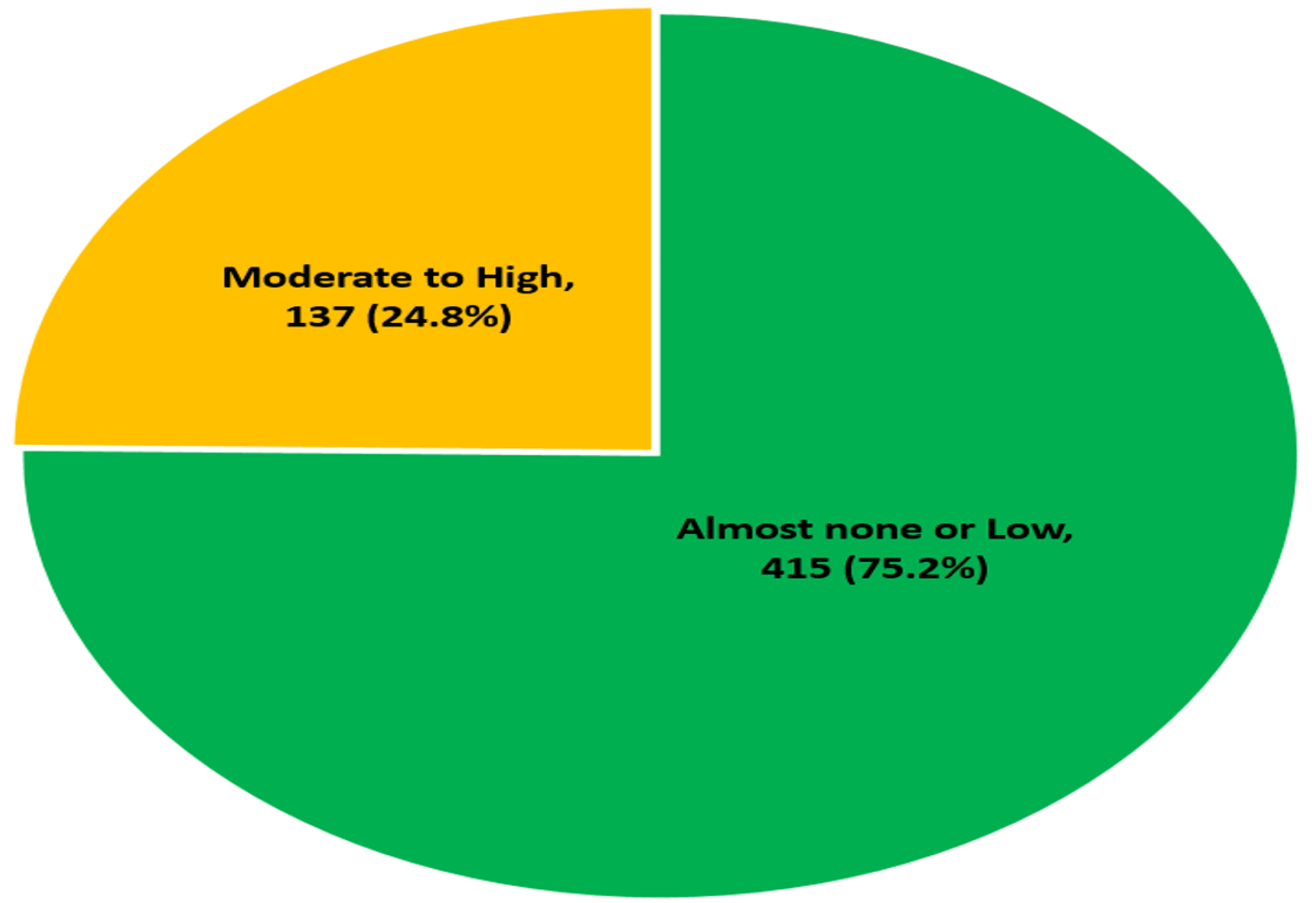
Vaccine hesitancy among healthcare professionals (n=552)

Respondents are categorized into two groups based on their levels of vaccine hesitancy: "Almost none or low" and "Moderate to high." The majority of healthcare professionals (415, 75.2%) reported almost none or low vaccine hesitancy, and a relatively low portion (137, 24.8%) reported moderate to high levels of vaccine hesitancy Figure 1.

The association between the level of vaccine hesitancy and the characteristics of workers, perceptions about vaccination/vaccines is presented in Table 4. No significant association was found between sex, age, education level, occupation, duration of work, and vaccine hesitancy. But workers who worked in governmental hospitals had significantly higher vaccine hesitancy (p=0.002), while Bahraini workers had significantly lower vaccine hesitancy compared to non-Bahraini workers (p=0.005). Moreover, participants who believed vaccines protect against diseases, protect vulnerable individuals, prevent epidemics, promote public health, and prevent long-term complications had a significant association with lower hesitancy (p < 0.001 for all).

**Table 4:** Association of level of vaccine hesitency and charactersitics of workers, and perception about vacination/vaccine (n=552) P-value by Chi-square test

		Vaccine Hesitancy Frequency (Percent)		P-values
		Almost none or Low	Moderate to High	
Sex	Male	75 (18.1%)	30 (21.9%)	0.323
	Female	340 (81.9%)	107 (78.1%)	
Age	20 – 30 years	129 (31.1%)	45 (32.8%)	0.762
	31 – 40 years	208 (50.1%)	65 (47.4%)	
	41 - 50 years	59 (14.2%)	23 (16.8%)	
	51 - 60 years	17 (4.1%)	3 (2.2%)	
	61 - 70 years	2 (0.5%)	1 (0.7%)	
Nationality	Bahraini	395 (95.2%)	121 (88.3%)	0.005
	Non-Bahraini	20 (4.8%)	16 (11.7%)	
Education	Diploma degree	67 (16.1%)	25 (18.2%)	0.585
	Bachelor degree	181 (43.6%)	67 (48.9%)	
	Master degree	47 (11.3%)	14 (10.2%)	
	Medical degree	108 (26%)	29 (21.2%)	
	Other	12 (2.9%)	2 (1.5%)	
Occupation	Doctor	158 (38.1%)	42 (30.7%)	0.283
	Nurse	183 (44.1%)	69 (50.4%)	
	Laboratory worker	74 (17.8%)	26 (19%)	
Duration of working	< 10 years	223 (53.7%)	76 (55.5%)	0.723
	> 10 years	192 (46.3%)	61 (44.5%)	
Institute	Primary Care	241 (58.1%)	59 (43.1%)	0.002
	Governmental hospitals	174 (41.9%)	78 (56.9%)	
Vaccines that healthcare workers should receive	Hepatitis B	412 (99.3%)	128 (93.4%)	<0.001
	Measles-mumps-rubella (MMR)	354 (85.3%)	92 (67.2%)	<0.001
	Influenza	361 (87%)	97 (70.8%)	<0.001
	Tetanus-diphtheria (Td)	355 (85.5%)	89 (65%)	<0.001
	Varicella	246 (59.3%)	47 (34.3%)	<0.001
	Meningococcal conjugate	335 (80.7%)	88 (64.2%)	<0.001
	SARS-CoV-2	308 (74.2%)	86 (62.8%)	0.01
Perception about vaccines	Protect us from diseases	375 (90.4%)	97 (70.8%)	<0.001
	Protect vulnerable individuals	292 (70.4%)	73 (53.3%)	<0.001
	Prevent epidemics and pandemics from occurring	331 (79.8%)	82 (59.9%)	<0.001
	Promote public health	326 (78.6%)	79 (57.7%)	<0.001
	Prevent long-term complications of diseases	286 (68.9%)	53 (38.7%)	<0.001
	Don't think they are important	6 (1.4%)	9 (6.6%)	0.01

Multivariate analysis of the association between vaccine hesitancy and perception about vaccination/vaccine is presented in Table 5. Multivariate analysis revealed that workers who worked in government hospitals were significantly associated with moderate to higher vaccine hesitancy (RR = 1.8), while Bahraini workers had significantly lower vaccine hesitancy compared to non-Bahraini workers (RR = 0.383). According to multivariate analysis, workers who believed vaccines protect against diseases (RR = 0.388), prevent epidemics (RR = 0.562), promote public health (RR = 0.527), and prevent long-term complications (RR = 0.366) had significantly lower hesitancy. Those who did not think vaccines were important had significantly higher hesitancy (RR = 3.464).

**Table 5:** Multivariate analysis of association between vaccine hesitancy and perception about vaccination/vaccines (n=552) ǂ Almost none or low, RR=Relative risk, 95% CI = Confidence Interval, P-value by logistic regression

		Moderate to high vaccine hesitancy^ǂ^		
		RR	95% C.I.	P-values
Nationality	(Ref. Non-Bahraini)	0.383	0.19 – 0.76	0.01
Institute	(Ref. Primary care)	1.8	1.24 – 2.7	0.002
Vaccines that healthcare workers should receive	Hepatitis B	0.192	0.05 - 0.76	0.019
	Measles-mumps-rubella (MMR)	0.466	0.28 - 0.77	0.003
	Influenza	0.464	0.27 - 0.81	0.007
	Tetanus-diphtheria (Td)	0.519	0.3 - 0.91	0.023
	Varicella	0.578	0.36 - 0.94	0.028
	Meningococcal conjugate	0.61	0.38 - 0.98	0.043
	SARS-CoV-2	0.586	0.39 - 0.88	0.011
Perception about vaccines	Protect us from diseases	0.388	0.23 - 0.67	0.001
	Protect vulnerable individuals	0.856	0.52 - 1.41	0.542
	Prevent epidemics and pandemics from occurring	0.562	0.34 - 0.94	0.029
	Promote public health	0.527	0.32 - 0.87	0.012
	Prevent long-term complications of diseases	0.366	0.22 - 0.6	<0.001
	Don't think they are important	3.464	1.04 - 11.52	0.043

## Discussion

The debate over the importance of healthcare workers' immunization has heated up since the SARS-CoV-2 pandemic. The WHO recommends that all health workers should be fully vaccinated with any additional vaccines as per the national schedules for adult immunization in use in their country and have documented proof of immunity or immunization [16]. Health care professionals with an updated vaccination card play an important role in reducing the risk of nosocomial spread of infections, especially to vulnerable patients, and reducing the risks of outbreaks related to different vaccine-preventable diseases on numerous occasions [18]. Our study showed a wide variation in vaccine coverage for the different types of vaccines that are recommended for HCPs in the Kingdom of Bahrain. Hepatitis B vaccine and varicella vaccine coverage need serious attention from the decision-makers. The WHO also recommends that a policy decision must be made about whether health worker vaccination will be voluntary or mandatory or a combination of both [16]. The majority (79.5%) of participants in our study supported mandatory occupational vaccination. Supporting mandatory vaccination was found to be significantly associated with vaccine uptake among HCPs; however, mandatory vaccination can cause ethical dilemmas and not be culturally acceptable everywhere [16, 18, 19, 20, 21, 22].

Health care professionals’ knowledge, attitudes, hesitancy, experiences, and behaviors related to vaccination were analyzed in many studies. An overall finding of one of the literature reviews that included 185 articles was that knowledge about particular vaccines, their efficacy, and their safety helped HCPs build their own confidence in vaccines and their willingness to recommend vaccines to others [17]. Moreover, vaccine confidence varies across nations, as revealed by a previous study [23]. A recent study published in France found that 98-99% of HCPs had confidence in the ability of vaccination to protect against infectious diseases [24]. In our study, 75% of the participants believed that vaccines are safe, 85.5% believed that vaccines protect against infectious diseases, and 58.7% of HCPs always encouraged patients to receive vaccines. These findings reveal that HCPs in the Kingdom of Bahrain believe in the importance of vaccination. The main limitation of this study is the low response rate during recruitment among medical doctors; therefore, many specialties were not included in this study. The low response rate is not surprising given the demanding nature of this cohort’s occupation

Vaccination hesitancy among health care professionals worldwide has become a topic of growing concern in recent years. While reviewing the literature, there are many different parameters used to calculate vaccine hesitancy among HCPs across all regions. However, standardization and unified calculation do not exist. In our study, we created our own measurement tool based on the questions of the survey, and we found that almost all the HCPs in our study reported that they were strongly favorable to vaccination, with 75.2% of them having none or low vaccine hesitancy. In one of the studies in France that included 1,795 HCPs, 93.7% were found to be strongly favorable to vaccination, and 42.2% (95% CI = 39.8-44.6) showed moderate vaccine hesitancy. Also, they found that the prevalence of vaccine hesitancy was lower among infectious disease specialists (12.3%; 95 CI = 6.7-21.3) [8]. Unfortunately, because of the low sample size of different specialties in our study, we couldn’t compare vaccine hesitancy among different specialties.

In our study, workers who worked in government hospitals and non-Bahraini workers had significantly higher vaccine hesitancy compared to the others. Many factors were reported to be associated with vaccine hesitancy, but there was no universal algorithm; the independent and relative strength of influence of each factor is complex and context-specific, varying across time, place, and type of vaccine [25, 26]. Vaccine hesitancy was linked during the SARS-CoV-2 pandemic to a fear of vaccine-related adverse side effects in many published studies [27-30]. In our study, long-term side effects were found to be the top concern about vaccination in our participants (48.4%).

The WHO recommends that courses and training about immunology and the importance of vaccines should be conducted for HCPs [16]. In our study, lack of training was found to be a major problem that needed to be solved, especially since the majority of the participants were willing to be educated regarding vaccination/vaccines.

## Conclusions

In the era of vaccine hesitancy, there is an urgent need to develop an approach to reduce and address apprehensions towards vaccinations. It is important to create policies that include strategies to improve confidence in vaccine uptake that are adapted based on the social, cultural, and economic context of the country or region.

Taken together, our results underscore the urgent need to address vaccine hesitancy in HCPs and to conduct more qualitative studies to better understand the various determinants underlying vaccine hesitancy among HCPs, especially in our region. Moreover, the implementation of policies to check and record the vaccination status of HCPs and make sure that they have received the required training will play a major role in increasing the vaccine uptake and reducing hesitancy.
